# Blood Pressure Variability (BPV) as a Novel Digital Biomarker of Multisystem Risk and Diagnostic Insight: Measurement, Mechanisms, and Emerging Artificial Intelligence Methods

**DOI:** 10.3390/biomedicines14020317

**Published:** 2026-01-30

**Authors:** Lakshmi Sree Pugalenthi, Sidhartha Gautam Senapati, Jay Gohri, Hema Latha Anam, Hritik Madan, Adi Arora, Avni Arora, Jieun Lee, Gayathri Yerrapragada, Poonguzhali Elangovan, Mohammed Naveed Shariff, Thangeswaran Natarajan, Jayarajasekaran Janarthanan, Shreshta Agarwal, Shiva Sankari Karuppiah, Divyanshi Sood, Swetha Rapolu, Vivek N. Iyer, Scott A. Helgeson, Shivaram P. Arunachalam

**Affiliations:** 1 Department of Internal Medicine, Mercy Catholic Medical Center, Darby, PA 19023, USA; 2 Department of Internal Medicine, Texas Tech University Health Sciences Center, El Paso, TX 79905, USA; 3 Digital Engineering & Artificial Intelligence Laboratory (DEAL), Mayo Clinic, Jacksonville, FL 32224, USAlee.jieun@mayo.edu (J.L.); thangeswarann@gmail.com (T.N.); agarwal.shreshta98@gmail.com (S.A.);; 4 Department of Neuroscience, Virginia Tech, Blacksburg, VA 24061, USA; 5 Department of Internal Medicine, UChealth Parkview Medical Center, Pueblo, CO 81005, USA; 6 Medical Gastroenterology, AIG Hospitals, Hyderabad 500032, Telangana, India; 7 Division of Pulmonary Medicine, Department of Medicine, Mayo Clinic, Rochester, MN 55901, USA; 8 Division of Pulmonary Medicine, Department of Medicine, Mayo Clinic, Jacksonville, FL 32224, USA; 9 Department of Critical Care Medicine, Mayo Clinic, Jacksonville, FL 32224, USA

**Keywords:** visit-to-visit variability, ambulatory blood pressure monitoring, vascular stiffness, autonomic dysfunction, wearable monitoring

## Abstract

Hypertension has been traditionally known to be highlighted by mean blood pressure; however, emerging evidence exhibits that blood pressure variability (BPV), including short-term, day-to-day, and visit-to-visit fluctuations can have an implication across multiple body systems. Elevated BPV reflects repetitive hemodynamic stress, affecting the physiologic hemostasis contributing to vascular injury and end organ damage. This narrative review is a compilation of recent evidence on the prognostic value of BPV, explained by pathophysiology, various devices with its measurement approaches, and, essentially, the clinical implication of BPV and the use of such devices utilizing artificial intelligence. A comprehensive literature search across PubMed, Cochrane Library, Scopus, and Web of Science were conducted, focusing on observational studies, cohorts, randomized trials, and meta-analyses. Higher BPV has been associated with an increased risk of cardiovascular mortality, stroke, coronary events, and heart failure, the progression of chronic kidney disease, cognitive decline, and preeclampsia, among other end organ damage, despite mean blood pressure. The various pathophysiologic mechanisms include autonomic dysregulation, arterial stiffness, endothelial dysfunction, circadian rhythm alteration, and systemic inflammation, which result in vascular remodeling and multisystem damage. Antihypertensive medications such as calcium channel blockers and renin–angiotensin–aldosterone system inhibitors seem to reduce BPV; randomized trials have not specifically investigated their BPV-reducing effects. The aim of this review is to highlight that BPV is a dynamic marker of multisystem risk, and question how various AI-based devices can aid continuous BPV monitoring and patient specific risk stratification.

## 1. Introduction

The management of hypertension is traditionally based on average blood pressure, which has been supported by compelling evidence that shows cardiovascular complications and even death. Current guidelines recommend treating blood pressure with stable ranges of values, but overlook the fluctuations that occur in blood pressure over shorter periods of time, such as seconds, hours, days, or years. Such changes in blood pressure are now termed as blood pressure variability (BPV) and are now increasingly recognized as clinically important due to their multisystem effect. While some BPV is noted as a physiologic response to daily needs, persistent and excessive changes in BPV have been identifies as a risk factor for organ damage and can be associated with poor outcomes, highlighting why relying only on average BP readings is insufficient [1].

Relying solely on mean blood pressure can potentially miss the impact of swings, which can place the body under stress. For example, two different people with the same mean blood pressure would face different risks based on their BPV. Several reviews and meta-analyses have demonstrated that visit-to-visit BPV can strongly predict cardiovascular events and death, irrespective of their mean blood pressures [2]. The long-term benefits of some blood pressure medications are suspected to come from their effects on lowering BP variability rather than lowering the averages alone [3].

In this review, diagnostic insight refers to BPV as a biomarker that reveals the disruptions in the harmony of the body’s homeostatic mechanisms and multisystem dysregulation. These are key factors that play a role include the autonomic nervous system, which regulates sympathetic activity and baroreflexes to handle any changes; however, its disruption is common in conditions like diabetes or sleep apnea, which can cause an increase in BPV [4]. The renin–angiotensin–aldosterone system contributes by causing blood vessel constriction and fluid retention, thereby worsening the variability in blood pressure in people with hypertension [4]. Arteries with increased stiffness are less pliable, making them unable to absorb changes in pressure, leading to even greater fluctuations and promoting inflammation [4].

BPV has a significant impact across the different organ systems. Studies have identified repetitive pressure changes on the kidney filters, which possibly contribute to the rapid progression of chronic kidney disease [5], while unstable blood flow to the brain or vascular damage could be tied to cognitive decline and higher chances of dementia [6]. Larger population studies have demonstrated that such variability has the ability to affect health markers like glucose, cholesterol, and body mass index, thereby increasing the risk of death [7]. This suggests that BPV can cause various bodily systems to fail and potentially can be a sign of a larger pattern homeostatic instability, leading to widespread issues [7].

BPV is categorized into (1) very short-term BPV (beat-to-beat), (2) short-term BPV (within 24 h; typically assessed by ambulatory monitoring), and (3) long-term BPV (day-to-day or visit-to-visit variability across weeks to years), consistent with European Society of Hypertension recommendations.

The limitations of BPV lie in its measurement and its use in everyday care. The difficulty in measuring BPV arises from differences in devices, the frequency of readings, and environmental factors such as stress or seasons [8]. Interpreting results requires established limits for abnormal levels, and there remains debate over whether BPV is associated with such problems or whether they simply occur simultaneously [9,10]. Although calcium channel blockers have the potential to reduce variability, larger trials are required to assess their effect on BPV and improve outcomes, thereby informing guideline revisions and emphasizing their indication [4].

This review aims to compile and integrate the latest evidence on BPV as a multisystem risk indicator. We will discuss the different categories of BPV, the pathophysiologic mechanisms leading to BPV, how it ties to various multisystem complications, ways to measure BPV, and, finally, the integration of BPV into current clinical practice, risk assessment, and management.

This manuscript is a narrative review that aims to bring together the available epidemiologic, mechanistic, and technological evidence related to BPV. It does not present any original models or data. It seeks to conceptualize BPV as a prognostic marker of multisystem risk and complications, and to highlight current devices that can aid in the measurement of BPV and its potential integration into clinical practice.

This review uses the European Society of Hypertension’s definitions to explain blood pressure variability (BPV). Short-term BPV covers changes from one heartbeat to the next and up to 24 h, usually measured with ambulatory blood pressure monitoring (ABPM). Mid-term BPV describes day-to-day changes over several days. Long-term BPV refers to differences between visits over weeks, months, or years. These definitions are used throughout the manuscript to keep the discussion clear and consistent.

This review adds several important points to the research on blood pressure variability (BPV). First, it uses clear and consistent definitions for short-, mid-, and long-term BPV, bringing together different perspectives to fill the gaps left by earlier studies. Second, it reviews the evidence linking BPV to health outcomes, making a clear distinction between well-known links—especially in heart, kidney, and brain health—and newer or less certain findings in areas like hormones, mental health, blood, and the immune system. Third, the review looks at both traditional and newer ways to measure BPV, including wearable devices and artificial intelligence, and discusses their limits and how close they are to being used in practice. Overall, the review shows that BPV is not just a side effect. It points to BPV as a sign of wider instability in the body and sums up what is known now and what still needs to be studied.

## 2. Materials and Methods

We carried out a narrative review using PubMed/MEDLINE, Scopus, Web of Science, and the Cochrane Library. The last search took place on 30 June 2025. Our search terms were “blood pressure variability,” “visit-to-visit variability,” “ambulatory blood pressure,” “beat-to-beat blood pressure,” “wearable blood pressure,” and “cuffless blood pressure monitoring.” We included peer-reviewed observational cohorts, randomized controlled trials, post hoc trial analyses, systematic reviews, and meta-analyses with human subjects published in English. We excluded case reports, animal-only studies, editorials, and non-peer-reviewed web sources. We did not use formal risk-of-bias scoring tools or PRISMA flow diagrams because this is a narrative review, not a systematic review or meta-analysis. We describe the strength of evidence qualitatively in the text and summarize it in tables. We did not perform any new quantitative analyses or pooled estimates. Table 1 summarizes the key characteristics, relative merits, and limitations of the study types included.

## 3. Mechanisms to Outcomes: An Integrated Review

Blood pressure variability, which describes the variation in blood pressure across different time periods and its potential to predict multisystem risk and events, has gained momentum over mean blood pressure, which cannot achieve the same. The three broad categories include: short-term (beat-to-beat, minute-to-minute, or within 24 h), mid-term (day-to-day or between days within a week), and long-term (visit-to-visit variability across weeks, months, or years). Accurate blood pressure measurement is the basis for clinical decision making and risk stratification [27]. Although office-based blood pressure measurements are widely used, their values are limited by single-point variability and white coat effects. This is improved by home blood pressure monitoring, which addresses the limitations of office blood pressure measurements by providing readings over several days in a comfortable setting, but it lacks continuous monitoring. Ambulatory blood pressure monitoring (ABPM) emerged as the most reliable method for short-term BPV measurement as it captures blood pressure at timed intervals (usually every 15–30 min) across 24 h and even during sleep [28]. This aids in the detection of diurnal patterns such as dipping and non-dipping, morning surges, and nocturnal hypertension. ABPM triumphs over office and home measurements, as it can also detect masked and white coat hypertension. Furthermore, ABPM-derived BPV is better associated with target organ damage, such as left ventricular hypertrophy and microalbuminuria, than office blood pressure measures [29]. Due to these advantages, ABPM is a valuable tool in both research and clinical practice for assessing dynamic blood pressure fluctuations and improving cardiovascular risk assessment.

**Figure 1:**
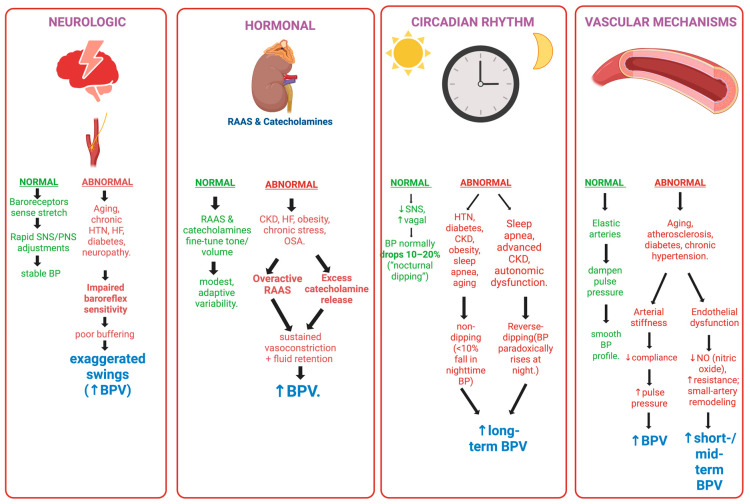
This schematic outlines the mechanistic pathways contributing to blood pressure variability (BPV) through neurologic, hormonal, circadian, and vascular regulation.

Blood pressure variability encompasses the dynamic fluctuations in blood pressure over different time scales. The BPV categories referenced here follow the standardized definitions introduced in Section 1. Such minuscule variations result from the interplay among the body’s neural, hormonal, vascular, and circadian regulatory systems, as depicted in Figure 1.

### 3.1. Neural Mechanisms

The autonomic nervous system (ANS) plays an integral role in short-term BPV through its sympathetic and parasympathetic branches. The sympathetic nervous system (SNS), when stimulated, releases catecholamines such as norepinephrine, leading to vasoconstriction, tachycardia, and increased myocardial contractility. This action can raise blood pressure, contributing to significant minute-to-minute fluctuations [29]. In contrast, the parasympathetic nervous system (PNS) exerts a counter effect, causing vasodilation and decreasing heart rate, thereby lowering BP.

The baroreflex is a critical feedback loop that monitors arterial pressure changes through specialized baroreceptors in the carotid sinus and the aortic arch. These receptors relay information to the brainstem, which then brings balance between the SNS and PNS activity to stabilize blood pressure. However, impaired baroreflex sensitivity in conditions like hypertension or heart failure can diminish its balancing effects and lead to uncontrolled BPV [29].

### 3.2. Hormonal Mechanisms

The renin–angiotensin–aldosterone system (RAAS) plays a key role in exerting its effects on both short- and long-term BPV. When decreased blood pressure or decreased volume is detected, the RAAS is activated, producing angiotensin II, a vasoconstrictor that increases vascular resistance and thereby raises BPV. Another component of RAAS, aldosterone, also contributes by retaining sodium and water and directly impacting blood volume and pressure [30], whereas catecholamines from the adrenal medulla enhance SNS-mediated BPV, especially during stressful scenarios. In conditions like hypertension or renal dysfunction, the RAAS is disrupted and, hence, exacerbates BPV by increasing vascular tone and altering fluid balance, leading to persistent BP fluctuations [31].

### 3.3. Vascular Mechanisms

Arterial stiffness significantly impacts BPV. Pathophysiologic processes such as atherosclerosis and chronic hypertension alter arterial elastic properties, leading to arterial stiffening. This decreased compliance reflects the dampened effects of pulsatile ejection of blood from the heart, resulting in pronounced pressure fluctuations and increased pulse pressure [2]. This mechanical limitation of the arteries culminates in higher systolic pressures and larger BP swings, thereby contributing to short-term BPV.

Furthermore, endothelial dysfunction, facilitated by decreased nitric oxide production, affects the blood vessels’ ability to vasodilate and regulate blood flow. This adds to the increased vascular resistance and elevated BPV. In hypertension, remodeling of small resistance vessels further amplifies these effects and thereby affects both short- and mid-term BPV [29].

### 3.4. Circadian Mechanisms

It has been detected that blood pressure exhibits a circadian rhythm primarily orchestrated by the suprachiasmatic nucleus in the hypothalamus. Physiologically, blood pressure has a nocturnal dip (10–20% decrease during sleep) due to reduced SNS activity and increased parasympathetic vagal tone [32]. This physiologic mechanism is in place to reduce the cardiovascular load during rest. However, if deviations occur that disrupt this mechanism, they can result in “non-dipping” (insufficient nocturnal BP reduction) or “reverse-dipping” (nocturnal BP elevation). Such deviations occur in conditions such as sleep apnea, diabetes, or chronic kidney disease. Such abnormal circadian patterns have been associated with increased long-term BPV and accelerated target organ damage [31].

### 3.5. Interplay: Autonomic Nervous System and Arterial Stiffness in BPV

The intertwined effects of ANS and arterial stiffness synergistically influence BPV. The ANS modulates heart rate, cardiac output, and systemic vascular resistance, and thereby regulates BP fluctuations. Conditions such as stress, obesity, or long-standing hypertension result in a state of chronic sympathetic overactivity and directly increase BPV through vasoconstriction and increased heart rate variability, whereas, in an aging population or those with pre-existing cardiovascular disease, it impairs the baroreflex system and thereby results in poorly buffered acute blood pressure changes, essentially turning into a marker for higher short-term BPV [29].

Arterial stiffness, measured by pulse wave velocity (PWV), is a marker of BPV. Stiffened arteries have decreased elasticity, resulting in higher systolic blood pressure and more pronounced BPV due to changes in stroke volume or peripheral resistance [2].

This phenomenon is particularly notable in older adults and individuals with advanced atherosclerosis, where increased arterial stiffness widens pulse pressure and affects short-term BPV. This exaggerated pulsatile stress contributes significantly to hemodynamic strain on vital target organs, including the heart and kidneys. Thus, while the ANS drives dynamic changes, arterial stiffness amplifies their impact concurrently.

## 4. Studies Linking BPV to Target End Organ Damage

A compilation of evidence from various studies shows that BPV is associated with different forms of organ damage, independent of mean blood pressure. These consistent, rapid fluctuations in blood pressure cause repetitive, damaging stress across the body’s systems.

### 4.1. Left Ventricular Hypertrophy (LVH)

Amodeo et al. demonstrated a significant independent association between higher 24 h ambulatory systolic BPV and the occurrence of LVH in hypertensive patients. High BPV creates a constant and repetitive hemodynamic stress on the left ventricle, causing it to work harder, resulting in pathologic myocardial remodeling and subsequent hypertrophy [30].

### 4.2. Endothelial Dysfunction

Parati et al. exhibited that short-term BPV correlates with endothelial dysfunction, typically assessed via flow-mediated dilation. The fluctuation caused in blood pressure induces increased shear stress on the vascular endothelium, which can decrease the production of the protective vasodilator nitric oxide and promote chronic vascular inflammation and endothelial damage [29].

### 4.3. Other Target Organ Damage

The effects of BPV extend beyond the heart and the vascular endothelium. Tai et al. reported that visit-to-visit BPV is a potent predictor of major cerebrovascular events, such as stroke, and coronary events [32]. Furthermore, high BPV is associated with progression of renal dysfunction, with studies describing its damage to the glomerulus in patients with chronic kidney disease [31].

## 5. BPV as a Predictor of Systemic Complications

BPV is a powerful and emerging predictor of adverse clinical outcomes across multiple organ systems, beyond traditional average blood pressure measurements. We have summarized the findings of various cohort studies, randomized controlled trials, and meta-analyses that provide supporting evidence.

### 5.1. Cardiovascular Complications

The ASCOT-BPLA trial [33] and the VALUE trial [11] established BPV as a strong and independent predictor of stroke. The ASCOT-BPLA study, in particular, demonstrated the benefits of amlodipine over atenolol in reducing stroke risk through its ability to reduce BPV, specifically the standard deviation of visit-to-visit systolic blood pressure. Meta-analyses show that higher BPV is associated with a significantly increased risk of both ischemic and hemorrhagic stroke [33].

The SPRINT trial [12] suggests that high BPV, especially short-term variability, is associated with a greater incidence of myocardial infarction. The constant changes in blood pressure and sympathetic tone are believed to increase myocardial oxygen demand and contribute to plaque instability, leading to rupture and thrombosis.

Increased BPV can also be a predictor of the incidence of heart failure. Long-term studies show that constant hemodynamic stress on the myocardium, leading to pathologic cardiac remodeling and LVH, is a key mechanism linking BPV to heart failure [13,14].

Meta-analyses of multiple observational studies and RCTs have noted BPV as a key predictor of all-cause and cardiovascular mortality, independent of average blood pressure levels [11,14]. This highlights its role as a marker of cardiovascular damage.

### 5.2. Renal Complications

Elevated BPV is associated with a faster decline in the estimated glomerular filtration rate (eGFR) and an increased risk of albuminuria in patients with and without pre-existing chronic kidney disease (CKD) [15]. This is due to pressure fluctuations in the renal vasculature, which damage the glomeruli, leading to proteinuria and worsening kidney function [5]. A systematic review and meta-analysis of 23 studies supported this association between BPV and CKD progression [5,34].

The successive damage caused by persistent BPV progresses to end-stage renal disease (ESRD). Longitudinal studies have shown that long-term elevated BPV is associated with a greater risk of requiring dialysis in patients with CKD, demonstrating its ability as a prognostic marker [15].

### 5.3. Neurological Complications

The association of increased BPV with accelerated cognitive decline and a high risk of developing dementia has been demonstrated by several prospective cohort studies [35,36]. BPV results in erratic blood flow and microvascular damage in the brain and is thought to be the underlying mechanism for the decline in executive function and memory. This association is prominent in vascular dementia, which is essentially due to damage to small vessels in the brain [34].

These mechanistic changes, which cause elevated BPV, are demonstrated by a greater burden of cerebral small vessel changes, including white matter hyperintensities and silent brain infarcts evident on neuroimaging [34]. A long-term study over 14 years demonstrated that visit-to-visit systolic BPV is associated with an increased progression of WMH volumes and a higher risk of incident lacunar infarcts [34].

### 5.4. Pulmonary Complications

The relationship between OSA and BPV is bidirectional. Sleep apnea disrupts the physiologic nocturnal equilibrium between SNS and PNS, amplifying the nocturnal blood pressure variations initiated by acute hypoxemia which activates the sympathetic nervous system [37]. These hypoxemia-induced sympathetic surges create significant blood pressure fluctuations. Such blood pressure surges experienced by patients during sleep correlate directly with the severity of oxygen desaturation [38]. It has been demonstrated that patients with severe OSA exhibit significantly higher nocturnal systolic and diastolic BPV compared to those with mild-to-moderate OSA. Severe OSA patients show increased nocturnal systolic BPV (12.1 ± 6.0 vs. 7.6 ± 4.3; *p* < 0.01) and diastolic BPV (10.5 ± 6.1 vs. 7.3 ± 4.0; *p* < 0.05) [39]. There is a sex-specific association with BPV due to autonomic dysfunction being more pronounced in female patients with OSA, especially those with elevated diastolic BPV compared to the controls [39].

There also lies a relationship between BPV and chronic obstructive lung disease (COPD). Pulmonary hypertension (PH) in COPD has diverse phenotypes, from end-stage COPD with moderate PH to pulmonary vascular phenotypes with severe PH [40]. The pathophysiology underlying this involves hypoxic pulmonary vasoconstriction, vascular remodeling, and endothelial dysfunction, which, in turn, affect blood pressure variability by altering cardiac output and venous return [41]. Acute hypoxemia is also associated with increased baroreceptor sensitivity, which in turn increases BPV [42]. Studies show that hypoxia also causes a compensatory increase in heart rate and contributes to the increased BPV in patients with chronic hypoxic conditions [42].

### 5.5. Immunological Complications

The link between BPV and systemic inflammation is an interplay of complex pathophysiologic mechanisms with significant clinical implications. Many inflammatory markers have notable associations with increased BPV, especially interleukin-6 (IL-6), CRP, and tumor necrosis factor-alpha (TNF-α) [43,44]. IL-6 has the greatest potential as a marker of BPV. In a large, diverse cohort of 5483 patients, baseline serum IL-6 was significantly associated with increased BPV, even after adjustment for other factors (odds ratio 1.49, 95% CI 1.28–1.74, and *p* < 0.001) [43]. This link remained even after accounting for other inflammatory markers, demonstrating IL-6′s role in BPV development. The Systemic Immune Inflammation Index (SII), which includes platelet, neutrophil, and lymphocyte counts, demonstrated a strong correlation with ambulatory BPV (r: 0.619, *p* < 0.05) [44]. SII has a sensitivity of 77% and specificity of 71% for predicting ABPV > 14, highlighting its prognostic ability to identify multisystem risks.

Patients with rheumatoid arthritis (RA) exhibit significantly higher visit-to-visit systolic BPV compared to non-RA subjects (13.8 ± 4.7 vs. 13.0 ± 5.2 mm Hg; *p* = 0.004) [45]. This increased variability is associated with adverse cardiovascular outcomes, with visit-to-visit systolic BPV showing an increased risk of cardiovascular events (hazard ratio 1.12 per 1 mm Hg increase; 95% CI 1.01–1.25) [45]. Noticeably, systolic BPV declined after RA diagnosis, reflecting improved disease management or treatment effects.

Patients with systemic erythematosus lupus (SLE) have been identified to have elevated diastolic BPV. Diastolic BPV ≥ 9 mm Hg is associated with increased cardiovascular event risk (relative risk 2.1, 95% CI 1.0–4.1, and *p* < 0.05) [46]. The relationship between SLE disease activity, corticosteroid use, and BPV creates a complex interplay in which the inflammatory processes themselves directly influence blood pressure variability [35,46]. The relationship between BPV and inflammation can be explained through cytokine-mediated endothelial dysfunction, SNS activation, and vascular remodeling [47,48,49]. TNF-α and IL-6 contribute to hypertension through their direct effects on renal and vascular functions, while chronic inflammation promotes arterial stiffness and impairs baroreceptor sensitivity [49,50].

### 5.6. Hematological Complications

The impact of BPV on the hematologic system is mediated by vascular shear stress and subsequent endothelial dysfunction. The high frictional force of blood flow on the vascular endothelial cells disrupts vascular homeostasis [51,52]. Fluctuations in blood pressure also cause variable shear stress patterns, resulting in disturbed endothelial and platelet function. The endothelium normally maintains anticoagulant properties through the production of prostacyclin and nitric oxide, which inhibit platelet aggregation [53,54]. However, increased BPV can impair these protective mechanisms. Studies demonstrate that endothelial dysfunction correlates with increased platelet reactivity, particularly in patients with stable coronary artery disease [55]. This relationship is explained by the decreased production of nitric oxide and prostacyclin, leading to increased platelet aggregation and a prothrombotic state [53].

Hypertension, including increased BPV, creates a prothrombotic state despite exposure to high vascular pressures [56,57]. This state, in turn, involves multiple mechanisms: chronic shearing stress and inflammation, which convert the normal anticoagulant endothelial surface into a procoagulant surface, which expresses tissue factor; fibrinolytic imbalance, as demonstrated by the altered balance between the tissue plasminogen activator and plasminogen activator inhibitor-1; and, finally, platelet activation, exhibited by enhanced platelet activation markers, including an increased mean platelet volume, platelet distribution width, and platelet hematocrit in hypertensive patients [58].

In sickle cell disease, BPV may contribute to vaso-occlusive crisis through various pathophysiologic mechanisms. The complex interaction between sickled erythrocytes and endothelial dysfunction creates conditions where blood pressure fluctuations could potentially trigger or exacerbate vaso-occlusive episodes [59,60,61]. The adhesion of the sickled cells to the endothelium, combined with inflammatory responses, creates a microenvironment where BPV-induced changes in shear stress and potentially influence sickle cell crisis frequency and severity.

In contrast, increased BPV is also associated with elevated bleeding risk, particularly in anticoagulated patients. An analysis of patients with atrial fibrillation on anticoagulation showed that those in the highest quartile of systolic BPV had a significantly increased major bleeding risk (OR 1.9; 95% CI 1.6–2.25) [62]. This highlights the complex relationship between thrombotic and hemorrhagic risks in patients with high BPV.

### 5.7. Endocrine Complications

Increased BPV can disrupt various endocrine pathways involving the hypothalamic paraventricular nucleus, the hypothalamic–pituitary–adrenal (HPA) axis, and the RAAS. The paraventricular nucleus integrates autonomic and neuroendocrine signals, coordinating sympathetic outflow and hormone release. BPV impairs the central secretion of CRH and ACTH, which in turn disrupts the normal diurnal cortisol pattern and diminishes the feedback sensitivity of the HPA axis [63,64,65,66].

Elevated BPV correlates with abnormal cortisol secretion characterized by the loss of normal circadian rhythm and episodic surges. This leads to prolonged HPA activation, manifesting as Cushing syndrome, which includes central obesity, glucose intolerance, and immunosuppression. As described earlier, BPV amplifies RAAS via episodic renin and aldosterone secretion, thereby resulting in sodium retention, volume expansion, and vascular remodeling, which, in turn, promote further BP fluctuations, LVH, and renal injury [63,64,67].

Hyperthyroidism increases BPV through increased cardiac output and decreased systemic vascular resistance, leading to arrhythmias, while hypothyroidism may blunt BPV and result in diastolic hypertension [63,64,68].

The bidirectional relationship between BPV and catecholamines is characterized by mutual amplification and feedback mechanisms. Norepinephrine and epinephrine from sympathetic nerve endings and the adrenal medulla raise BPV by causing increased vascular tone and cardiac output. In conditions such as stress, exercise, or pathologic states like pheochromocytoma, variations in BP occur, including paroxysmal hypertension and the loss of circadian BP patterns [69,70,71,72].

Baroreflex and central regulatory pathways intertwine when BP fluctuates, which in turn increases the sympathetic drive and catecholamine release to maintain BP stability. In hypertensive patients, elevated urine normetadrenaline correlates with increased systolic BPV, supporting the association between sympathetic activity and BPV [73].

A normal acute increase in blood pressure activates baroreceptors, resulting in the GABA-mediated inhibition of neurons releasing vasopressin. This baroreceptor-mediated inhibition is blunted in the setting of increased BPV, leading to persistently elevated vasopressin neuron firing and increased circulating vasopressin, even when blood pressure is high [74,75,76,77]. Hence, disrupted BPV can alter vasopressin secretion and impair water balance, contributing to nocturnal hypertension and hyponatremia, especially in the context of sleep disorders or heart failure [63,64].

Hyperparathyroidism is associated with increased BPV, possibly through hypercalcemia-induced vascular dysfunction, resulting in further persistent hypertension and vascular calcification [68].

Increased BPV is associated with increased insulin resistance and metabolic syndrome, which promotes atherosclerosis and thereby increases cardiovascular risk [63,64]. Increased BPV is associated with heightened sympathetic tone and stress, which in turn suppresses the GHRH from the hypothalamus and enhances somatostatin release, leading to reduced and blunted nocturnal GH secretion [63,68,78,79].

### 5.8. Psychiatric/Behavioral Outcomes

BPV can influence mental and behavioral systems. Elevated BPV is associated with the increased prevalence and severity of depressive symptoms, anxiety symptoms, and suicide risk. Numerous studies show that people with depression and generalized anxiety disorder (GAD), and panic disorder exhibit elevated short-term and long-term BPV compared to controls, irrespective of treatment with antihypertensive medications [80,81,82,83].

In elderly people, elevated diastolic BPV is independently linked to the heightened intensity of depressive symptoms, especially dysphoria, even after controlling for confounding factors such as baseline depression and medication usage [81]. Longitudinal studies have showed that GAD is associated with elevated systolic BPV across 8 years, supporting the impact of anxiety on BP regulation [82].

In contrast, younger individuals with an increased lifetime burden of depressive episodes predict elevated short-term BPV, suggesting that the incremental impact of depression may induce autonomic dysregulation early in life [83].

BPV is also independently linked to increased suicide mortality, which is particularly prominent in older individuals. The association between BPV and psychiatric outcomes is probably mediated by autonomic dysfunction, diminished baroreflex sensitivity, and endocrine dysregulation [1,84].

Although AI models frequently show acceptable performance in controlled environments, their accuracy is diminished in the presence of high intrasubject BPV and real-world conditions. Factors such as physiologic variability, signal noise, and nonstationary blood pressure dynamics limit generalizability and underscore the need for external validation in diverse hypertensive populations.

### 5.9. Pregnancy Complications

Increased BPV has been associated with adverse outcomes during pregnancy for both the mother and the fetus. It can result in gestational hypertension, preeclampsia, and severe hypertension in the mother, and its effects on fetal growth include the fetus being small for its gestational age or having a low birth weight [85].

Multiple cross-sectional studies have described that beat-to-beat BPV is elevated in pregnant women with preeclampsia in comparison to healthy pregnant women [86].

A prospective cohort study was able to conceptualize that beat-to-beat BPV is a feature of early pregnancy starting in first trimester and remains elevated after diagnosis into the second and third trimester, reflecting hemodynamic/autonomic dysregulation contributing to preeclampsia. Higher BPV is associated with aortic stiffness, measured by carotid pulse wave velocity (cfPWV), irrespective of the average BP. This aortic stiffness is theoretically thought to modulate the blood pressure variability seen in pregnancy although this is not fully understood [86].

## 6. BPV vs. Mean Blood Pressure as Predictors of Clinical Outcomes

The BPV categories referenced here follow the standardized definitions introduced in Section 1 [4,15,87,88]. Mean BP is the average BP (systolic, diastolic, or mean arterial pressure) at a given period, such as a clinic visit, a 24 h ambulatory monitoring, or numerous other visits. It is currently the primary parameter used for hypertension diagnosis and management [89,90].

BPV can serve as an independent predictor of multisystem risk and mortality, separate from mean blood pressure. Cohort studies and meta-analyses have shown that elevated long-term BPV is associated with an increased risk of all-cause mortality, cardiovascular mortality, stroke, and coronary events, even when controlling for mean blood pressure. The hazard ratio for all-cause mortality per standard deviation increase in long-term systolic BPV is roughly 1.15, comparable to traditional risk factors like cholesterol [2].

While mean blood pressure is the current principal modifiable risk factor for cardiovascular disease, BPV provides additional prognostic insights beyond mean BP. The ADVANCE-ON and ACCORD trials, along with various observational cohorts, demonstrate that BPV independently predicts outcomes regardless of the mean BP [91,92,93]. The impact of BPV is apparent even in patients with adequately managed mean BP, underscoring the independent significance of BPV [94]. Strong data also indicate that the negative effects of BPV are more significant in patients with a lower mean BPV [11].

## 7. BPV Metrics

BPV is quantified using standard deviation, the coefficient of variation, or variance, independent of the mean. The coefficient of variation (CV) is calculated as the SD divided by the mean blood pressure. The average real variability (ARV) is the mean absolute difference between consecutive readings, and better captures temporal instability. Reliable BPV measurement requires multiple readings: typically ≥20 for 24 h ambulatory BPV and ≥5–7 for visit-to-visit BPV [16].

Standard deviation and the coefficient of variation are common ways to analyze blood pressure from one visit to another or in clinical trials, but both depend on the average blood pressure. The average real variability (ARV) may be a better option for tracking changes over time, especially for short-term or ambulatory blood pressure. ARV is most useful when measurements are taken often and at regular intervals, but it is less reliable with only a few readings. The best measurement method depends on when the data is collected, how much data there is, and what the clinical goals are.

## 8. Pharmacologic vs. Non-Pharmacologic Modulation of BPV

### 8.1. Pharmacologic

Recent meta-analyses support greater BPV reduction with angiotensin-converting enzyme inhibitors (ACEi) and angiotensin receptor blockers (ARBs) compared to other antihypertensive medications. The ONTARGET trial demonstrated that telmisartan decreased visit-to-visit BPV by 12% compared with ramipril [95]. Amlodipine, a dihydropyridine calcium channel blocker, has consistently reduced BPV across multiple studies.

The ASCOT-BPLA post hoc analysis demonstrated that amlodipine decreased BPV by 23% compared with atenolol, contributing to its stroke-reducing benefits [95]. Traditional beta-blockers may increase BPV, whereas newer agents such as nebivolol demonstrate neutral or beneficial effects. This may be explained by its vasodilatory properties and its impact on arterial stiffness [95].

Although several antihypertensive classes reduce BPV, current evidence does not establish that BPV reduction itself mediates outcome benefit. To date, BPV has not been the primary therapeutic target in randomized trials, and observed benefits may reflect broader vascular or hemodynamic effects [96].

### 8.2. Non-Pharmacologic

The DASH diet, potassium (K+) supplementation, and salt restriction are known to control BPV [97]. Moderate-intensity workouts and isometric exercises reduce BPV [98]. Stress management, primarily through mindfulness and sleep hygiene, and a consistent sleep schedule, are associated with a reduction in BPV [99].

## 9. BPV as a Clinical Marker

Enhanced BPV is associated with increased multisystem morbidity and mortality, independent of the mean blood pressure. The key findings include the following:

1. Stroke Risk: Visit-to-visit BPV independently predicts stroke incidence (RR 1.15 per 1-SD increase in systolic BPV) [100].
2. Cognitive Decline: Higher BPV associates with accelerated cognitive deterioration and dementia risk [99].
3. Target Organ Damage: BPV correlates with left ventricular hypertrophy, carotid intima–media thickness, and albuminuria progression [101].

## 10. Artificial Intelligence and BPV

The accurate and continuous monitoring of BPV has been enhanced by advanced sensor technology and artificial intelligence (AI). Traditional ambulatory and office monitoring methods are limited by their ability to measure recordings only intermittently, often failing to detect high variability patterns. AI such as machine learning (ML) and deep learning (DL) can extract clinically significant features from continuous, high-volume data generated by wearable devices, thereby overcoming the limitations of ambulatory and office monitoring methods. Most digital and AI-based systems do not directly measure blood pressure variability. Instead, they first estimate beat-to-beat or intermittent blood pressure values from physiological signals, after which BPV metrics (e.g., SD, ARV, and CV) are derived computationally across time.

It is important to distinguish blood pressure estimation from blood pressure variability analysis. AI models are typically trained to estimate systolic and diastolic blood pressure from signals such as photoplethysmography (PPG), electrocardiography (ECG), impedance, or tonometry. Blood pressure variability is then derived by applying standard statistical metrics to these estimated BP values across predefined time windows. Wrist-worn devices use PPG and ECG to determine the pulse arrival time (PAT) and, in turn, to calculate the pulse transit time (PTT), which is inversely related with beat-to-beat BPV. This method requires frequent patient-specific calibration, which results in high inter-subject variability, a challenge AI must overcome.

Deep learning models such as recurrent neural networks (RNNs) and convolutional neural networks (CNNs) are now being strengthened to better capture the relationships among PPG, ECG, and invasive arterial blood pressure waveforms. These DL models are trained to analyze large volumes of clinical datasets and detect BPV patterns associated with multisystem risks that are missed by conventional measurement devices. AI is key to risk stratification, using not only the BPV metrics but also integrating other factors, such as patient demographics, to predict adverse events across body systems with high accuracy. The future lies in personalizing these AI models with techniques such as transfer learning, which can overcome the limitations of manual calibration, enabling continuous blood pressure estimation and the true detection of BPV.

AI is increasingly being used with home, ambulatory, and wearable devices to extract and analyze extensive data. Deep neural networks have demonstrated the capacity to interpret blood pressure variability over prolonged periods of up to 4 weeks and to integrate multiple datasets, including BP measurements and clinical factors. These models can be personalized to predict both mean BP and BPV, which is clinically significant given the association between BPV and multisystem risks [102].

**Figure 2:**
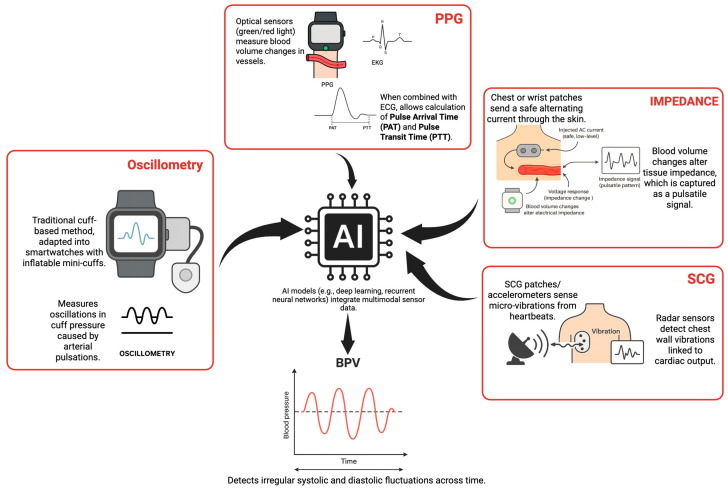
Overview of wearable sensor technologies and artificial intelligence (AI) integration for blood pressure variability (BPV) monitoring. Oscillometry, photoplethysmography (PPG), impedance, and seismocardiography (SCG) provide multimodal physiological signals that AI algorithms synthesize to detect BPV.

Wearable technology, such as wrist-worn devices and sensor-equipped wearables, utilizes methods such as oscillometry, photoplethysmography (PPG), seismocardiography (SCG), and bioimpedance, to assess and monitor BPV as depicted in Figure 2 [102].

Wrist-worn oscillometric devices are a condensed form of cuff-based methodology but have been known to frequently overestimate BP in normotensive individuals and underestimate BP in hypertensive patients, resulting in measurement errors relative to upper-arm blood pressure devices [102]. PPG-based wearables employ optical sensors to measure variations in blood volume in the vasculature and integrate PPG with ECG, as described above, to detect BPV. The drawback of these models is that their accuracy may diminish with significant intrasubject BPV or in practical use [17,18,103,104,105].

Bio-impedance wearables utilize sensors to assess variations in electrical impedance across the skin, indicating pulsatile blood flow. In some advanced devices, data quality and prediction accuracy are enhanced by utilizing both bioimpedance and PPG sensors. Machine learning techniques, such as random adaptive boosting, integrate multimodal data from multiple sensors to facilitate the continuous monitoring of blood pressure variability [18,19].

Recent advances in tonometry have led to wrist- and finger-worn devices that use implantation tonometry to capture arterial pressure waveforms. These devices detect continuous arterial pressure data without the use of an inflatable cuff. AI models, such as recurrent neural networks and deep learning, analyze various waveform morphologies to detect BP and its variability. Tonometry-based devices are better able to estimate BPV in ambulatory and lab settings, with low systolic BP errors [0.3 mmHg (SD ~9.8)] and 24 h averages within 1 mmHg of the reference values in young, normotensive individuals. Factors such as posture, calibration, and population diversity affect the accuracy of such devices and pose a challenge in real-world settings [19,20].

Contactless radar sensors and seismocardiography (SCG) are innovative and new technologies for the non-invasive monitoring of BPV. Radar systems detect microvibration over the chest wall caused by cardiac output and arterial pulsation, as reflected in pulse wave velocity and BP, and enhance AI-based data extraction. Studies with neural networks show root mean square errors of 3–9 mmHg for systolic BP, but these systems are not validated for clinical use yet. Chest-worn accelerometers or patch sensors, which combine SCG with PPG or ECG, facilitate cuffless BPV monitoring. AI algorithms are trained to support signal fusion, noise reduction, and the extraction of BP features from complex data. The limitations of radar- and SCG-based devices include their susceptibility to motion artifacts and the need for further research to overcome this and enable accurate BPV measurement in real-world situations [21,22,23,24,25].

Cuffless systems require initial calibration using conventional cuff measurements and may exhibit decreased accuracy over time or with changes in user characteristics [86].

Most AI-based BPV studies use CNNs to extract waveform features and RNNs for temporal dependency modeling. These models are simultaneously trained on PPG, ECG, and invasive arterial pressure datasets to predict beat-to-beat blood pressure and, essentially, derive BPV metrics [26].

The performance of these models is typically evaluated using mean absolute error (MAE), root mean square error (RMSE), Bland–Altman analysis, and compliance with international validation standards such as AAMI and ISO guidelines. External validation across different populations remains limited, underscoring the need for standardized benchmarking [106].

AI-based techniques enhance the incorporation of multimodal data, encompassing physiological, behavioral, and environmental elements, to augment the precision of BPV evaluation and controlling hypertension and its effects. To improve model interpretability and facilitate practical, patient-specific intervention planning, supportive AI techniques are being developed. This review does not report on original artificial intelligence model development. The AI approaches discussed herein are derived from prior studies and are summarized to illustrate emerging analytic strategies rather than validated clinical tools.

The integration of wearable and cuffless technologies with AI enables the continuous, non-invasive monitoring and detailed evaluation of BPV, as shown in Figure 2. However, the accuracy and clinical applicability of these innovative devices need additional validation.

Model performance is commonly evaluated using mean absolute error, root mean square error, Bland–Altman analysis, and alignment with AAMI or ISO standards. However, most studies rely on internal validation within limited cohorts. External validation across diverse populations and clinical states remains scarce, limiting the immediate clinical applicability of AI-derived BPV metrics.

This review does not implement or train artificial intelligence models directly. Instead, it summarizes and contextualizes existing machine learning and deep learning architectures—most commonly convolutional neural networks (CNNs) and recurrent neural networks (RNNs)—that have been applied in prior studies for blood pressure estimation and variability analysis using wearable and ambulatory data. Table 2 shows as summary of the wearable and cuffless blood pressure monitoring technologies used for blood pressure variability (BPV) assessment.

## 11. Limitations

Office and home blood pressure readings fail to capture BPV accurately. These methods exhibit low reproducibility and significant variability due to the number of measurements, inadequate monitoring duration, and user operational errors. AHA reports that office BPV does not predict cardiovascular outcomes or all-cause mortality [4,15,107]

The lack of diagnostic test thresholds or quantitative characteristics of BPV are not yet established; hence, its current role is to be interpreted as a tool in providing diagnostic insight into systemic risks.

A lack of standardization in the measurement of BPV, including standard deviation (SD), the coefficient of variation, average real variability, and measurement obstacles, necessitates repeated assessments over time, the decreased reproducibility of short-term BPV, and the inaccuracy or absence of validation for novel cuffless and wearable devices. Office BPV is notably unreliable and lacks a correlation with its results. All of these factors make comparisons and clinical applicability difficult [87,88,108].

Several wearable and cuffless devices demonstrate systematic measurement bias, including the overestimation of blood pressure in normotensive individuals and underestimation in hypertensive patients. Accuracy is further affected by posture, motion artifacts, skin tone, vascular stiffness, and calibration drift. These limitations introduce error into BPV estimation, particularly during ambulatory and real-world monitoring.

In clinical practice, BPV does not have enough valid data to guide therapy. Although BPV has been shown to increase multi-systemic effects, as discussed in our text, RCTs have not shown that treatments targeting BPV improve outcomes. Long-acting CCB and thiazide diuretics may, however, lower BPV, but no RCTs show that this reduces multisystem complications and events [87,88].

The deterring factors for ACC, AHA, and the European Society of Hypertension in recommending BPV assessment and targeted treatment are the insufficient data and the lack of standardization. Significant gaps include a lack of RCTs evaluating BPV-guided treatment, a lack of standardization of measurement, and the clinical value of the new monitoring devices [90].

## 12. Discussion

BPV is a key prognostic marker of multisystem complications such as cardiovascular disease, stroke, coronary heart disease, heart failure, chronic renal disease, and dementia, among others. It can predict overall mortality independent of mean blood pressure. This close association is supported by numerous cohort studies and meta-analyses and is considered significant, comparable to traditional risk factors such as cholesterol [2,15,89,109,110]. Table 3 synthesizes these findings by grading the strength of evidence across organ systems, allowing differentiation between established prognostic relationships and emerging associative signals.

BPV demonstrates a strong and consistent independent association with cardiovascular, renal, and cerebrovascular outcomes. Associations with the endocrine, psychiatric, hematologic, and immunological domains are supported primarily by observational and mechanistic evidence and should be considered as emerging evidence rather than as causative. This relationship remains clinically significant even after adjusting for mean blood pressure, highlighting BPV’s ability to reveal pathophysiological processes not indicated by average values alone [2,4,87,89]. An integrated summary of BPV associations across organ systems is provided in Table 3.

The underlying mechanisms of BPV’s detrimental effects are multifaceted and include disrupted autonomic pathways, arterial stiffness, vascular endothelial damage, and a heightened inflammatory response. All of these processes result in increased hemodynamic stress, disrupt homeostasis, and expedite end organ damage. This evidence confirms that BPV is an indicator of instability in multisystem homeostasis [88,89,111,112].

Evidence from BPV-tailored treatment indicates that antihypertensives such as CCBs and RAAS inhibitors are more effective at lowering BPV than other antihypertensives and thereby improve outcomes, as demonstrated by large trials such as ASCOT-BPPLA and ONTARGET. Non-pharmacological therapies such as dietary modifications with the DASH diet, potassium (K+) supplementation, salt restriction, regular aerobic exercise, stress alleviation, and sleep enhancement may improve BPV [113,114]. However, these approaches have not been rigorously studied in randomized trials with defined BPV goals.

Future research must focus on three areas: (1) standard definitions, metrics, and measurement protocols for BPV; (2) validating novel wearable and cuffless technologies against established methodologies; and (3) larger randomized control trials to confirm if targeted BPV reduction leads to enhanced multisystem outcomes. Moreover, integrating BPV into management algorithms through explainable AI can enhance individualized treatment strategies for such patients [15,88,89,106,107,115,116].

The principal contribution of this review lies in that it reframes BPV from being a secondary hemodynamic phenomenon to being a dynamic marker of systemic homeostatic instability, while outlining how artificial intelligence-based analytics may enable continuous BPV assessment and personalized risk prediction.

This review consolidates evidence supporting BPV as a prognostic marker of multisystem risk, harmonizes BPV definitions and metrics, distinguishes BPV reduction from outcome benefit, and critically evaluates emerging digital and AI-based monitoring approaches. By integrating mechanistic, epidemiologic, and technological perspectives, this work provides a structured framework for future BPV-focused research and clinical translation. At present, AI-derived BPV metrics should not be used for direct clinical decision making. Their potential role lies in longitudinal risk stratification, trend monitoring, and hypothesis generation. Prospective studies are required to determine whether AI-guided BPV assessment improves patient prognosis or informs therapeutic adjustment. By clearly distinguishing prognostic association from diagnostic utility, BPV reduction from outcome mediation, and BP estimation from BPV derivation, this review addresses prior conceptual ambiguities and provides a standardized framework for future research. Accordingly, AI-derived BPV measures should currently be considered investigational and should not be used as standalone tools for clinical decision making.

## 13. Conclusions

BPV is a dynamic marker of multisystem homeostatic instability, with strong evidence supporting its prognostic role in cardiovascular, renal, and neurological outcomes, and emerging associative evidence in other organ systems. The original contribution of this work lies in integrating mechanistic insights, clinical evidence, and emerging digital monitoring technologies, while clearly identifying current limitations. By distinguishing association from causality and BPV reduction from outcome benefit, this review provides a balanced framework for future research aimed at validating BPV-guided strategies and AI-enabled monitoring in real-world clinical practice.

## Figures and Tables

**Table 1:** Overview of literature included in the narrative review.

Study Type	BPV Time Scale	Strengths	Limitations	Key References
**Observational cohorts**	Visit-to-visit	Large sample size; long follow-up	Residual confounding; association only	[2,5,11,12,13,14,15]
**Randomized trials (post hoc)**	Visit-to-visit	Controlled BP lowering	BPV not primary target	[11,12,13]
**Meta-analyses**	Short- and long-term	Pooled effect estimates	Heterogeneity in BPV metrics	[2,5,16]
**Wearable/AI studies**	Short-term	Continuous data capture	Limited validation; small cohorts	[17,18,19,20,21,22,23,24,25,26]

**Table 2:** Wearable and cuffless technologies for blood pressure variability assessment, including acquired signals, reference standards, analytic models, performance metrics, and validation status.

Device Type	Signal(s)	Ground Truth	Model Type	Metrics	External Validation
**Wrist PPG**	PPG ± ECG	ABPM/A-line	CNN/RNN	MAE, RMSE	Limited
**Tonometry**	Pressure waveform	Invasive BP	CNN	Bland–Altman	No
**Bioimpedance**	Impedance + PPG	ABPM	ML ensemble	MAE	No

**Table 3:** Strength of evidence was classified as strong (consistent associations across multiple large cohorts and meta-analyses), moderate (supported by fewer cohorts or less consistent findings), or emerging (primarily observational or mechanistic studies without longitudinal validation).

Organ System	BPV Type	Outcome	Strength of Evidence	Interpretation
**Cardiovascular**	Visit-to-visit	Stroke, MI, HF	Strong	Independent prognostic association
**Renal**	Long-term	CKD progression	Moderate–strong	Accelerated decline in eGFR
**Neurological**	Visit-to-visit	Dementia, WMH	Moderate	Vascular-mediated injury
**Endocrine**	Short-term	HPA axis dysregulation	Emerging	Associative
**Psychiatric**	Long-term	Depression, suicide risk	Emerging	Autonomic dysfunction

## Data Availability

This review was based on publicly available academic literature databases.
